# Histopathological Changes as a Predictor of Fatal Outcome in Patients With Drug‐Induced Liver Injury

**DOI:** 10.1111/liv.70182

**Published:** 2025-06-13

**Authors:** Sabine Weber, Franziska Erhardt, Jens Neumann, Julian Allgeier, Didem Saka, Nirali Donga, Izabel Mircheva, Rochell Balakumar, Christian M. Lange, Alexander L. Gerbes

**Affiliations:** ^1^ Department of Medicine II LMU Klinikum Munich Germany; ^2^ Institute of Pathology, Medical Faculty Ludwig‐Maximilians‐Universität Munich Germany

**Abbreviations**
AST: aspartate aminotransferase
DILI: drug‐induced liver injury
LT: liver transplantation
NPV: negative predictive value
OR: odds ratio
PPV: positive predictive value
RUCAM: Roussel Uclaf Causality Assessment Method
TBIL: total bilirubin

We read with great interest the recently published review by David E. Kleiner on the role of liver biopsy in the diagnosis and management of drug‐induced liver injury (DILI) [1]. Kleiner points out that the value of liver biopsy does not only lie in the support of the DILI diagnosis and the exclusion of alternative causes of liver injury, but that liver biopsy can also provide prognostic information.

Since patients with a higher mortality risk should be evaluated for liver transplantation (LT) as rapidly as possible, the reliable identification of patients at risk for a fatal outcome early in the course of the disease is of utmost importance. We therefore aimed to identify histopathological features that are associated with need for LT or death in DILI patients. In this regard, we analysed the clinical and histological data of 136 DILI patients from our prospectively collected cohort of patients with acute liver injury and potential drug‐related cause, who were enrolled between 2013 and 2022 at our centre at the LMU Klinikum, Munich, Germany. The procedures were in accordance with the Helsinki Declaration of 1975, as revised in 2013 and the study protocol was approved by the local ethics committee (Project number 55‐13). Written informed consent was obtained from all subjects. The details of the study have been described in detail elsewhere [2]. Out of 483 patients included in our study, histopathological specimens were available for review in 212 patients. A thorough hepatological work‐up was performed and DILI was diagnosed in 136 of the 212 patients. DILI diagnosis was based on clinical and laboratory findings, the Roussel Uclaf Causality Assessment Method (RUCAM) score, causality assessment by the supervising physician, an expert opinion causality assessment process and upon long‐term follow‐up. Liver tissue was obtained by percutaneous liver biopsy (91.1%), while 2.2% received a transjugular liver biopsy and in 6.7% the histological analysis was performed in the liver explant. Histopathological reports were extracted from the patients' medical records in a standardised manner based on a pre‐defined catalogue. Severe necrosis was defined as confluent necrosis or liver dystrophy. Lipofuscinosis was defined as cytoplasmatic inclusion of lipofuscin in the hepatocytes. Any amount of lipofuscin detected in hepatocytes was rated as positive.

Twenty‐three of the 136 DILI patients had a fatal outcome (defined as death or LT; 16.9%), comprised of 14 (10.3%) patients who underwent LT and 9 (6.6%) patients who died. Comparison of clinical characteristics revealed no significant differences regarding age, sex, concomitant liver or autoimmune diseases nor pattern of liver injury between patients with a fatal or a more favourable outcome (Table 1). The main culprit drugs in patients with a fatal outcome were non‐steroidal anti‐inflammatory drugs (52.2%), antimicrobial drugs (13.0%), immune modulating drugs (13.0%) and herbal and dietary supplements (8.7%). Unsurprisingly, DILI patients with a fatal outcome had higher aminotransferases, total bilirubin (TBIL) and INR values at the time of DILI recognition. With regards to histopathological features, higher rates of severe necrosis and ductular proliferation were seen in patients with a fatal outcome (severe necrosis: 56.5% vs. 15.9%, *p* < 0.001; ductular proliferation: 73.9% vs. 18.6%, *p* < 0.001). In addition, there was a tendency towards a lower rate of lipofuscinosis in patients with a fatal outcome; however, this difference was not statistically significant (4.3% vs. 21.2%, *p* = 0.057, Table 1).

**TABLE 1:** Baseline characteristics of DILI patients with a favourable versus a fatal outcome.

	Favourable outcome (*n* = 113)	Fatal outcome^a^ (*n* = 23)	*p*
Clinical and laboratory parameters			
Age (years)	50 (18–84)	49 (19–80)	0.506
Body mass index (kg/m^2^)	24.4 (17.6–37.9)	24.6 (17.0–34.7)	0.953
Female gender	67 (59.3%)	13 (56.5%)	0.806
Concomitant autoimmune disorder	33 (29.2%)	7 (39.4%)	0.906
Pre‐existing liver disease	9 (8.0%)	3 (13.0%)	0.434
Culprit drugs			
NSAID	41 (36.3%)	12 (52.2%)	
Antimicrobial drugs	14 (12.4%)	3 (13.0%)	0.938
Immune modulators	16 (14.2%)	3 (13.0%)	
Herbal and dietary supplements	12 (10.6%)	2 (8.7%)	
Others	30 (26.5%)	3 (13.0%)	
*R* value at onset^b^	14.2 (0.2–54.0)	24.9 (0.3–83.1)	0.064
Pattern of liver injury based on *R* value^a^			
Hepatocellular	86 (76.1%)	19 (82.6%)	0.142
Mixed	15 (13.3%)	0 (0.0%)	
Cholestatic	12 (10.6%)	4 (17.6%)	
ALT onset (xULN)	24.0 (0.8–138.5)	45.4 (1.2–120.1)	**0.017** *
AST onset (xULN)	16.0 (0.6–92.5)	42.2 (1.8–202.2)	**< 0.001** *
ALP onset (xULN)	1.8 (0.5–21.0)	2.0 (1.0–11.6)	0.367
TBIL onset (xULN)	3.6 (0.2–41.5)	14.8 (1.4–29.2)	**< 0.001** *
INR onset	1.1 (0.8–4.6)	2.1 (1.0–5.3)	**< 0.001**
Hy's law positivity	82 (72.6%)	21 (91.3%)	0.056
Histopathological patterns			
Fibrosis	58 (51.3%)	13 (56.5%)	0.649
Inflammatory infiltrates	89 (94.7%)	23 (100%)	0.258
Interface hepatitis	53 (46.9%)	7 (30.4%)	0.147
Plasma cells	54 (47.8%)	9 (39.1%)	0.448
Lymphocytes	93 (82.3%)	19 (82.6%)	0.972
Neutrophils	48 (42.5%)	12 (52.2%)	0.393
Eosinophilic cells	64 (56.6%)	10 (43.5%)	0.248
Cholestasis	50 (44.2%)	13 (56.5%)	0.282
Severe necrosis	18 (15.9%)	13 (56.5%)	**< 0.001** *
Steatosis	28 (24.8%)	3 (13.0%)	0.221
Lipofuscinosis	24 (21.2%)	1 (4.3%)	0.057
Ballooning of hepatocytes	19 (16.8%)	6 (26.1%)	0.295
Hepatic sinusoidal dilatation	17 (15.0%)	2 (8.7%)	0.423
Ductular proliferation	21 (18.6%)	17 (73.9%)	**< 0.001** *

*Note:* Categorical variables are presented as number and percentage, *n* (%). Continuous variables are presented as median (range).

Abbreviations: ALP, alkaline phosphatase; ALT, alanine aminotransferase; AST, aspartate aminotransferase; DILI, drug‐induced liver injury; INR, international normalised ratio; NSAID, non‐steroidal anti‐inflammatory drugs; TBIL, total bilirubin; ULN, upper limit of normal.

^a^ Fatal outcome was defined as liver transplantation or death.

^b^ The *R* value is defined as: (ALT/ULN)/(ALP/ULN), with *R* ≥ 5 defining a hepatocellular, *R* ≤ 2 a cholestatic and 2 < *R* < 5 a mixed type injury.

* Bold numbers indicate a statistical significance (*p* ≤ 0.05).

We then performed an univariate logistic regression analysis, which revealed that the only histopathological features significantly associated with fatal outcome were severe necrosis and ductular proliferation (Table S1). In addition, the lack of lipofuscinosis also showed a trend towards a higher probability of a fatal outcome. Next, a stepwise backward logistic binary regression analysis was performed to identify a histopathological prediction model for fatal outcome in DILI. Included in this multivariate analysis were all histopathological features with *p* < 0.200 in univariate analysis. Table 2A shows that a model comprised of the absence of lipofuscinosis together with the presence of severe necrosis and ductular proliferation best predicted a fatal outcome in DILI patients. Overall accuracy of these histological model for the prediction of a fatal outcome was 89.7% (sensitivity 47.8%, specificity 98.2%), while the positive and negative predictive values (PPV and NPV) were 84.6% and 90.2%. Within this model, the highest association with a fatal outcome was seen for severe necrosis (odds ratio [OR] 5.55, *p* = 0.003) and ductular proliferation (OR 10.30, *p* < 0.001). Interestingly, the incorporation of baseline laboratory parameters that were significantly associated with DILI outcome in univariate logistic regression analysis could further enhance the prognostic accuracy: A model comprised of aspartate aminotransferase (AST), TBIL and INR at baseline together with the presence of ductular reaction and severe necrosis had an accuracy of 91.8% for the prediction of a fatal outcome at a sensitivity of 60.9% and a specificity of 98.2% (Table 2B). The highest association with a fatal outcome again was observed for ductular reaction (OR 5.26, *p* = 0.012; Table 2B).

**TABLE 2:** Logistic regression analysis for fatal outcome.

	Multivariate logistic regression		
	OR	95% CI	*p*
(A) Histological parameters^a^			
Severe necrosis	**5.550**	**1.805–17.061**	**0.003** *
Lipofuscinosis	0.143	0.015–1.376	0.092
Ductular proliferation	**10.304**	**3.374–31.471**	**< 0.001** *
(B) Laboratory and histological parameters^b^			
AST onset	1.0253	0.998–1.050	0.076
TBIL onset	**1.103**	**1.022–1.189**	**0.012** *
INR onset	**2.888**	**1.148–7.266**	**0.024** *
Severe necrosis	2.183	0.570–8.361	0.255
Ductular proliferation	**5.256**	**1.446–19.107**	**0.012** *

*Note:* Shown are the odds ratios (OR) and the 95% confidence intervals (CI) for histological features with regards to a fatal outcome in DILI patients established by logistic regression analysis. Variables considered in the multivariate logistic regression analysis were parameters with *p* < 0.200 in univariate analysis. Fatal outcome was defined as death or liver transplantation.

Abbreviations: AST, aspartate aminotransferase; CI, confidence interval; DILI, drug‐induced liver injury; INR, international normalised ratio; OR, odds ratio; TBIL, total bilirubin.

^a^ The binomial logistic regression model was statistically significant, *χ* ^2^(5) = 39.083, *p* < 0.001.

^b^ The binomial logistic regression model was statistically significant, *χ* ^2^(5) = 56.391, *p* < 0.001.

* Bold numbers indicate a statistical significance (*p* ≤ 0.05).

In conclusion, we could show that higher transaminases, TBIL and INR at DILI onset were associated with an increased risk for death or need for LT. Strikingly, histopathological features alone and especially in combination with AST, INR and TBIL had a high prognostic power. In order to provide distinctive features that can support the decision to evaluate DILI patients for high urgency LT, we developed a predictive model comprised of three histopathological features: The absence of lipofuscinosis in combination with severe necrosis and ductular proliferation could predict a fatal outcome with an outstanding accuracy of 89.7% and an extraordinarily high specificity of 98.2%, PPV of 84.6% and NPV of 90.2%. The histological model therefore performed better than for any other prognostic scores published on the prediction of acute liver failure in DILI [3, 4, 5]. A possible explanation for the correlation between higher severity and ductular proliferation is that in fulminant acute liver failure with insufficient regeneration of hepatocytes ductular reaction is the predominant type of progenitor cell reaction [6]. The association of lipofuscinosis with milder DILI on the other hand remains to be elucidated. It can be speculated that, since lipofuscinosis is associated with mitochondrial damage [7], hepatic lipofuscin is only observed in the case of a partial depletion of mitochondrial function. Our findings underline the importance liver biopsy plays in the evaluation of the severity of liver injury and the prognosis of the disease evolution. Liver biopsy can potentially aid in the decision making whether to proceed with a LT or not as it was recently highlighted by a case report on liver injury caused by green tea extract [8]. Nevertheless, liver biopsy should always be conducted with caution in the setting of severe liver injury which is often associated with coagulopathy and a possible risk of severe bleeding. In the setting of acute liver failure transjugular liver biopsy should be preferred in case of sufficient expertise. Our study has limitations, in particular the retrospective design of the histological data extraction. However, while the association of extensive necrosis and ductular reaction with a more severe liver injury and fatal outcome has been described before [6, 9, 10], to the best of knowledge this is the first report to show an association between lipofuscinosis and a less severe form of DILI. Thus, we can support the findings demonstrated by Kleiner that liver biopsy has a high value when it comes to evaluating the severity of liver injury and predicting outcome in DILI patients [1].

## Author Contributions

Conceptualisation: S.W.; methodology: S.W. and A.L.G.; software: S.W. and F.E.; investigation: S.W., F.E., J.A., D.S., N.D., I.M., R.B., J.N. and C.M.L.; formal analysis: S.W., F.E. and J.N.; validation: S.W., F.E. and A.L.G.; resources: S.W., J.A., C.M.L. and A.L.G.; data curation: S.W.; writing – original draft: S.W.; writing – review and editing: S.W., F.E., J.N., C.M.L. and A.L.G.; visualisation: S.W., F.E. and J.N.; supervision: S.W. and A.L.G.; project administration: S.W. and A.L.G.; funding acquisition: S.W. and A.L.G. All authors approved the final version of the manuscript.

## Ethics Statement

The study protocol conforms to the ethical guidelines of the Declaration of Helsinki and was approved by the ethics committee of the Faculty of Medicine, LMU Munich (Project Number 55‐13).

## Consent

Written informed consent was obtained from each participant.

## Conflicts of Interest

The authors declare no conflicts of interest.

## Supporting information


Table S1

## Data Availability

All data generated or analysed during this study are included in this article. Further enquiries can be directed to the corresponding author.
