# Supplementary material for: Histopathological Changes as a Predictor of Fatal Outcome in Patients With Drug‐Induced Liver Injury
Source: Liver Int. 2025 Jun 13;45(7):e70182. doi: 10.1111/liv.70182 (PMC12163973; doi:10.1111/liv.70182)
Supplement: Supplementary file 1 — Table S1 [file LIV-45-0-s001.docx]

**Suppl. Table 1 Univariate Logistic regression analysis for fatal outcome**

|  | **OR** | **(95 % CI)** | **p** |
| --- | --- | --- | --- |
| **AST onset** | 1.038 | 1.018-1.059 | **<0.001*** |
| **ALT onset** | 1.018 | 1.004-1.032 | **0.009*** |
| **ALP onset** | 1.046 | 0.885-1.235 | 0.600 |
| **TBIL onset** | 1.107 | 1.046-1.172 | **<0.001*** |
| **INR onset** | 10.177 | 3.251-31.863 | **<0.001*** |
| **Fibrosis** | 1.233 | (0.500-3.042) | 0.649 |
| **Interface hepatitis** | 0.495 | (0.189-1.296) | 0.147 |
| **Plasma cells** | 0.702 | (0.281-1.754) | 0.448 |
| **Lymphocytes** | 1.022 | (0.313-3.329) | 0.972 |
| **Neutrophils** | 1.477 | (0.601-3.630) | 0.393 |
| **Eosinophils** | 0.589 | (0.238-1.455) | 0.248 |
| **Cholestasis** | 1.638 | (0.663-4.045) | 0.282 |
| **Necrosis** | 1.824 | (0.629-5.291) | 0.264 |
| **Severe necrosis** | 6.861 | (2.611-18.030) | **<0.001*** |
| **Steatosis** | 0.455 | (0.126-1.648) | 0.221 |
| **Lipofuscinosis** | 0.169 | (0.022-1.315) | 0.057 |
| **Ballooning of hepatocytes** | 1.746 | (0.609-5.006) | 0.295 |
| **Hepatic sinusoidal dilatation** | 0.538 | (0.115-2.507) | 0.423 |
| **Ductular proliferation** | 12.413 | (4.368-32.275) | **<0.001*** |

Shown are the odds ratios (OR) and the 95 % confidence intervals (CI) for histological features with regards to a fatal outcome in DILI patients established by univariate risk analysis. * shows a statistical significance (p≤0.05).

Abbreviations: ALP: Alkaline phosphatase; ALT: Alanine aminotransferase; AST: Aspartate aminotransferase; CI: Confidence interval; DILI: Drug-induced liver injury; INR: International normalized ratio; OR: Odds ratio; TBIL: Total bilirubin.
